# Consumers’ Willingness to Pay for Food with Information on Animal Welfare, Lean Meat Essence Detection, and Traceability

**DOI:** 10.3390/ijerph16193616

**Published:** 2019-09-26

**Authors:** Lingling Xu, Xixi Yang, Linhai Wu, Xiujuan Chen, Lu Chen, Fu-Sheng Tsai

**Affiliations:** 1 Institute for Food Safety Risk Management, School of Business, Jiangnan University, Wuxi 214122, China; 8383800028@jiangnan.edu.cn (L.X.); 6180906013@stu.jiangnan.edu.cn (X.Y.); wlh6799@jiangnan.edu.cn (L.W.); xjchen@jiangnan.edu.cn (X.C.); 2 School of Humanity and Law, Northeast Agricultural University, Haerbin 150030, China; 3 Department of Business Administration, Cheng Shiu University, Kaohsiung 83347, Taiwan; tsaifs@gcloud.csu.edu.tw; 4 Center for Environmental Toxin and Emerging-Contaminant Research, Cheng Shiu University, Kaohsiung 83347, Taiwan; 5 Super Micro Mass Research and Technology Center, Cheng Shiu University, Kaohsiung 83347, Taiwan

**Keywords:** consumer, willingness to pay, pork, real choice experiment, China

## Abstract

Amid high-profile food scares, health concerns and threats of information imperfection and asymmetry, the Chinese pork industry faces increasing demands from consumers for assurances regarding quality and production methods in both the domestic and export markets. Using a real choice experiment (RCE), 316 consumers in Wuxi, located in China’s Jiangsu Province, were randomly surveyed to examine the impact of various factors (e.g., traceability, lean meat essence testing, animal welfare, appearance, and price) on consumers’ preference and willingness to pay (WTP) for pork products. A random parameter logit model was estimated, and the results show that having a traceable code is the second important factor after price for consumers, corresponding to a WTP of 4.76 yuan per catty, followed by a bright red appearance, a national stocking density standard of animal welfare, and detected no lean meat essence, corresponding to a WTP of more than 2 yuan per catty. In addition, there is a complementary interrelationship between a traceable code and a bright red appearance, detected no lean meat essence, and a national stocking density standard of animal welfare. The results concerning the latent class model (LCM) indicate that 56.9% of consumers are “quality-focused” consumers who are willing to pay a high price for traceable code, detected no lean meat essence, a national stocking density standard of animal welfare, and bright red appearance attributes. A further 28.1% are “price-sensitive” consumers who pay significant attention to the price, and the price that they pay for each product is meagre. The consumers with “preference combination attributes” attach greater value to interaction attributes, such as a traceable code combined with detected no lean meat essence or a bright red appearance and detected no lean meat essence combined with a national stocking density standard of animal welfare or a bright red appearance, accounting for 15% of consumers. The government should improve the traceability system, increase the intensity of lean meat essence testing, promote the welfare level of pigs, and promote public education and publicity on pork quality and safety attributes. Meanwhile, enterprises can formulate “differentiated” pork products, according to different consumer groups, and appropriately increase prices, according to production costs, in order to meet the requirements for pork quality and safety for consumers.

## 1. Introduction

China is a big producer and consumer of pork. However, there have been serious incidents relating to pork quality and safety. The most common food safety incidents in China, from 2006 to 2015, were related to meat and meat products. In the supply chain, involving pig breeding and slaughtering and the processing of pork, the number of food safety incidents to which the pig breeding sector is exposed ranked first. The food safety risks in the pig breeding industry are mainly related to the illegal use of feeds that add lean meat essence, a poor breeding environment, and the spread of disease and increase in the number of sick pigs. These pork quality and safety risks greatly affect consumers’ confidence and purchase choices [1]. How to improve consumer confidence has become a joint project of the government and producers. Lancaster’s [2] consumer utility theory holds that the utility of a consumer stems from the attributes of the commodity, rather than the commodity itself, which means that the value of the commodity is essentially the sum of the value of each attribute or characteristic of the commodity. In the food industry, Hobbs’ [3] study noted that food attributes constitute a very rich concept. In addition to the price and other commodity attributes, food attributes include physical food attributes, such as the flavor and nutrients, as well as production methods, animal welfare, environmental standards, presence of genetically modified ingredients, and other processing attributes related to food production. For meat products, in addition to the price, appearance, and nutrition attributes, food attributes can also include traceability, animal welfare, certificate of origin, quality inspection, environmental certification labels, and other additional information attributes that indicate the product quality and safety level [4,5]. Steenkamp, J.B.E.M. found that quality perception is regarded as an overall unidimensional evaluative judgment. It is a higher-level abstraction instead of a specific product attribute based on consumers’ perception of a product and quality attributes [6]. As for the form of labeling, information concerning the quality attributes of pork—indicating the elimination or reduction of food safety risks—is presented to consumers, which can differentiate products, reduce information asymmetry in the market, improve pork quality and safety, and help consumers make purchasing decisions [7,8].

**Figure 1:**
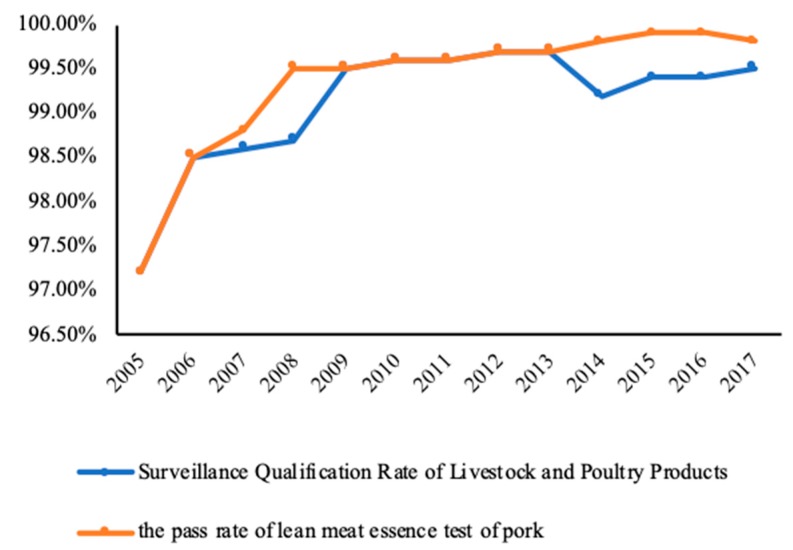
Qualification rate of the lean meat essence contaminant monitoring of livestock and poultry products in China, from 2005–2017.

Lean meat essence is a generic term for a class of drugs in China which includes adrenal nerve stimulants, such as clenbuterol, ractopamine, salbutamol, salbutamol sulfate, and terbutaline [9]. According to the Bulletin of the Ministry of Agriculture of China, No. 193 and 235, the detection of lean meat essence is restricted to livestock products sold on the market. Adding lean meat essence to pig feed can accelerate the fat metabolism and transformation of pigs, resulting in an increase in the lean rate of the pig and thus promoting the growth rate of the pig, reducing the feed amount, and shortening the feeding period. This helps pork producers to obtain more economic benefits [10]. However, lean meat essence is a non-protein hormone, which is heat resistant. The long-term consumption of feed containing lean meat essence may cause residues in pigs’ tissues, especially in internal organs. Consequently, the long-term consumption of pork or relevant products containing lean meat essence may directly or indirectly endanger human health [11]. For example, the intake of lean meat essence may cause chromosomal aberrations and induce malignant tumors and lean meat essence may increase the risk of hypertension, heart disease, hyperthyroidism, prostatic hypertrophy, or even death [12]. Figure 1 shows that, since 2005 in China, the rate of routine qualified monitoring of lean meat essence has remained at a high level. In recent years, China has been exposed to a new type of lean meat essence, mainly including olaquindox, cyproheptadine, and clonidine, which are not easily detected by the existing techniques and have more serious effects on human health. Thus, the prevention of “lean meat essence” still faces severe challenges.

In addition to the lean meat essence incident, there were 1615 quality and safety incidents between 2006 and 2015 in which China’s sick and dead pork entered the market, and from 2018 to 2019, large-scale African swine fever (ASF) infection has occurred in China, with 104 cases of ASF reported in 25 provinces. The poor pig breeding environment and unhealthy feeding practices, such as feeding pigs with swill, may be the major causes of the large number of sick or dead pigs and ASF. However, ensuring pigs’ welfare during the feeding process can reduce the incidence of epidemics by treating the source [13]. Many studies report that safeguarding animal welfare can improve meat quality and, consequently, food safety. For example, Velarde et al. [14] found that animal welfare can improve meat quality and safety, improve production efficiency, and reduce environmental pollution. It is a necessary condition for promoting the sustainable development of the economy, resources and environment. Gregory and Grandin [15] found that animal suffering is associated with animal disease and, thus, also with pharmacological treatments that are harmful to humans. On the other hand, animals reared under free range conditions with high welfare standards are seen as healthier animals, which will become higher-quality products. Harper and Henson [16] found that animal welfare and environmentally friendly labels are indicators of food safety and quality because food safety is often linked to food production methods. The United States, European Union, and other developed countries or regions attach great importance to animal welfare and have formed a mature legal protection system. However, so far, China has only two laws relating to poultry welfare, namely, “Farm Animal Welfare Requirements: Pig”, released in 2014, and “General Principles for the Evaluation of Animal Welfare”, released in 2017. In addition, the economy of China, with a large population, is developing rapidly and, correspondingly, the market demand for pork is growing fast. However, since China does not have mandatory laws and reasonable processing standards relating to pigs’ welfare, most pork producers or enterprises concentrate on maximizing economic benefits, but do not pay much attention to the welfare of pigs [17].

Generally, Chinese consumers are eager to obtain accurate information about the quality and safety of pork, such as the feed and the environment relating to the pig breeding process. In order to satisfy consumers’ demand for information and ensure the safety of pork, the Chinese Ministry of Agriculture and the Ministry of Commerce implemented a pilot program involving a traceability system for pig breeding in 2001. This requires that, in pilot cities, to ensure traceability of pork products, all information relating to the quality and safety of pork, such as the unitization of feed, vaccines, and veterinary drugs in the main processes of pork production (e.g., breeding, slaughtering, and marketing), as well as information on producers and quarantine officers at all stages of use, inspection, and quarantine, should be comprehensively recorded [18]. However, after nearly two decades of effort, the traceability system of pork has not yet covered the entire country of China. This is likely because the traceability information of pork has not yet met consumers’ requirements. Furthermore, the traceable pork purchased by consumers in the real pork market can only be traced back to the slaughter and processing stages. Information providing a pork traceability code is limited to information on, for example, the origin of the pork, name of the producer, who slaughtered the pig and sold the pork, and qualified inspection and quarantine information [19]. It does not fully display information on, for example, animal welfare and the result of lean meat essence testing during the pig breeding process, which consumers are concerned about [18], and the traceable query machine in the marketing process is almost ineffective.

To solve the quality and safety issue of pork in China, it is necessary to further improve the information disclosure system on pork quality and safety and promote the quality and safety attribute system on pork to meet consumers’ demands. This is an unavoidable challenge. However, comprehensive information may not necessarily help consumers make purchasing decisions [6]. In this paper, we use the real choice experiment method to elicit consumer preferences for different information attributes. To prevent consumer fatigue and improve the quality of collected data, when scholars use the choice experiment to study consumers’ preferences relating to product attributes, the number of attributes generally does not exceed six [20,21]. Based on the major food safety risks in China’s pork supply chain and the reality of the pork traceability system in China, this paper aims to study Chinese consumers’ preferences and willingness to pay (WTP) relating to animal welfare, lean meat essence test, traceability, and appearance attributes of pork products. There have been a lot of studies on consumers’ preference relating to traceability information [18,19,22,23,24,25,26,27,28,29,30], animal welfare [21,31,32,33,34,35,36,37], hormones or antibiotics [5,38,39,40,41,42], appearance [22,43,44,45,46,47,48], and other attributes of meat products in the United States, Britain, Germany, Spain, Australia, and other countries. In China, the largest developing country, due to the imbalanced economic and social development, the relative vastness of the region, the diversity of the food culture, and the complexity of the food safety issues, consumers’ preferences relating to the properties of meat products are specific to China. However, there have been quite a few studies on Chinese consumers’ preferences relating to animal welfare information, which can be found in [34,35]. These limited studies generally define animal welfare attributes as unpicked growth, feed with guaranteed quality, etc. This definition has not been subdivided into detailed criteria. In addition, no literature has reported Chinese consumers’ preference relating to the lean meat essence attributes of meat products. Therefore, based on the previous studies and special conditions in China, this paper sets lean meat essence detection, animal welfare, traceability, appearance, and price attributes, and studies the utility and value of these attributes of pork products for consumers. This could provide a basis for the decision-making of enterprises and the government in the management of the production of pork, with different quality and safety attributes, thereby improving the quality and safety of pork in China.

## 2. Research Methods and Experimental Design

### 2.1. Experimental Method

The experimental methods used in previous studies on consumers’ preferences relating to foods are mainly divided into two categories, namely, hypothetical methods and non-hypothetical methods. The contingent valuation method (CVM), conjoint analysis (CA), and choice experiment (CE) are hypothetical methods. While these experimental methods have different advantages, they are hypothetical and do not set the experiments in a real market environment. Since hypothetical methods are conducted in a hypothetical market environment, these methods would lead to hypothetical biases, where participants of a study knowingly or unknowingly misrepresent their preferences and WTP, thus leading to a distortion of the experimental results [49]. On the contrary, by carrying out an incentive compatible actual transaction with actual money and simulating a real market environment by providing actual products, non-hypothetical experiments such as experimental auctions (EA) and the real choice experiment (RCE) may effectively reduce the distortion of the results obtained through hypothetical experiments [50]. Therefore, both EA and RCE have become common methods for assessing consumers’ preferences in many countries [51]. However, compared to RCE, EA is often limited by its more complicated operation procedures and higher expense [3]. Real choice experiments, where the consumers’ choices have real consequences for how much money they can take home, are referred to as involving real economic incentives. A benefit of real economic incentives is that it makes it costly for the consumers to portray themselves as more altruistic than they really are, and this is likely to reduce the size of the social desirability bias. In real choice experiments, real markets are simulated by letting participants make a series of choices between products with posted prices. Each choice situation is referred to as a scenario, and the scenarios differ with respect to the product and price combinations included in them. Real economic incentives are introduced by randomly drawing one of the choice scenarios as binding and letting the participants buy their chosen product in that scenario at its posted price. In this kind of experiment, it is in the participants’ own interests to choose their preferred alternative in each scenario, and their incentives to reveal true preferences are relatively transparent. Therefore, RCE was used in the present study to elicit Chinese consumers’ preferences relating to pork with different attributes. A random parameter logit (RPL) model and latent class model (LCM) are used to estimate respondents’ WTP for different attributes and check for the presence of heterogeneity among the respondents.

### 2.2. The Setting of Attributes and Their Corresponding Levels

In this paper, pork hindquarters were selected as the product object of valuation, and information on their attributes and corresponding levels, which are presented in Table 1, were established based on previous studies and the national conditions of China. The attributes considered in this paper are traceability, lean meat essence testing, animal welfare, appearance, and price. Because of consumers’ preference for a label displaying food quality and safety information [7], this paper lists lean meat essence testing information and animal welfare information as two separate attributes of pork, both of which are displayed as labels. Based on the hazards relating to lean meat essence, many countries have clear regulations or laws prohibiting the use of lean meat essence. Clenbuterol, salbutamol, salbutamol sulfate, and terbutaline are highly toxic and harmful to human health, and their use is prohibited all over the world. Ractopamine has a relatively lower level of toxicity, compared to the other types of lean meat essence, and is easily decomposed and excreted by the human body. However, the residue of ractopamine, after in vivo metabolism, mainly accumulates in the liver of pigs. In some Western countries (e.g., the United States), pigs’ organs (e.g., the liver) are normally not consumed. Thus, ractopamine is allowed to be added to pigs’ feed in those countries. In China, since pig breeding is still dominated by small-scale farming in individual families, it is difficult for governments to regulate the use of hormones, such as lean meat essence, in feed. In addition, pigs’ organs are quite popular in China. Thus, the Chinese Ministry of Agriculture has banned lean meat essence since 1999. In the livestock industry, a rectification campaign which particularly focused on lean meat essence was carried out randomly. However, lean meat essence incidence is still found in pork meat. Foreign literature research mainly studied consumers’ preferences relating to the information on the utilization of hormones or antibiotics in meat products, as mentioned above. Due to the differences in national conditions, the illegal use and harmful influence on human health of lean meat essence drugs is particularly serious in China. This paper sets the lean meat essence test attribute with two corresponding levels. One level is “detected no lean meat essence”, if the test result shows that the pork does not contain lean meat essence, and the other level is no information about lean meat essence.

It is common knowledge that the animal welfare protection of pigs has not received widespread attention in China. For example, in the processing of pig breeding, the stocking density is too high, the environment of hog farming is unsanitary, and the feeding method has not been standardized. These factors result in an increase in piglet mortality and, also, cause a decrease in the reproductive rate of sows. Consequently, the injury frequency is significantly increased, and this is followed by a chronic stress response, weakened physique, and deceased resistance. This makes pigs susceptible to illness and reduces the meat quality [52]. Meanwhile, to minimize the losses caused by the illness and death of pigs, farmers increase the use of antibiotics or other drugs, thus facilitating a vicious circle. This causes hidden dangers to the quality and safety of pork. The animal welfare attribute set by Blokhuis et al. [53], Viegas et al. [40], and Velarde et al. [14] includes reducing the stocking density of pigs and providing comfortable and freely moveable accommodation. Denver et al. [33] set the animal welfare attribute based on the indoor hog farming space of finished pigs, with three levels: standard (the legally specified indoor area is at least 0.65 m^2^ per pig), medium (finisher pigs have 30% more space, that is, at least 0.85 m^2^ per pig), and high (finisher pigs have 100% more space, that is, at least 1.3 m^2^ per pig). One of the most serious issues relating to pigs’ welfare in China is the stocking density, which is too high and does not meet the national requirements. According to the animal welfare attributes of breeding density and their level settings (mentioned above), this paper sets two animal welfare attribute levels: a certain level of stocking density, which is in line with the national standards, and no stocking density (see Table 1).

In addition, Wu et al. [27] indicated that traceability systems should involve essential safety factors such as farming, slaughtering, transportation, and marketing, and the existing literature showed that consumers prefer complete traceability information. Therefore, in this paper, two settings are provided for the traceability attribute: a traceable code displaying complete breeding, slaughtering, transportation marketing information, or no traceable code (see Table 1). The appearance attribute mainly considers the freshness of pork. Pork sold in the market will have a different color because of the different storage time. The shorter the storage time, the fresher the pork will be, and the color is bright red. As storage time increases, freshness decreases, and the color becomes light red. People usually judge the freshness of pork by its appearance. Thus, we only set the color levels according to the appearance attribute [44,54]: bright red for very fresh and light red for generally fresh. The price of regular pork hindquarters was set at 12 yuan/500 g, based on a pre-survey of supermarkets, such as Vanguard and Tianhui supermarkets in Wuxi China in July 2018. Wu et al. [27] used the real choice experimental method, finding that consumers can accept traceable pork price rise of 20%–30%. Therefore, the price of pig hindquarters, with different levels of quality and safety attribute labels, is floating on the benchmark price of 12 yuan/catty, set at four levels of 12, 14, 16, and 18 yuan/catty.

### 2.3. Questionnaire Design

**Figure 2:**
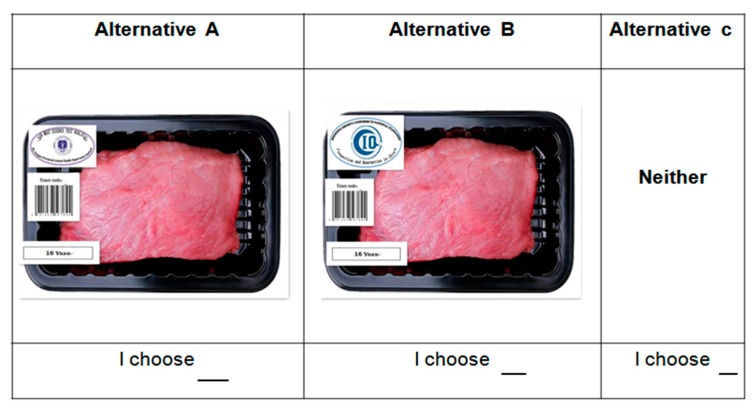
Example of a choice task for a package of 0.5 kg of pork hindquarters.

The questionnaire for this experiment is divided into three parts, including individual characteristics, consumers’ perception of each attribute, and the real choice experiment. Drawing on So and Kuhfeld [55] and Kuhfeld [56], a statistical analysis system (SAS; SAS Institute, Cary, NC, USA) was used to design the selection of the real choice experiment. In general, a full factorial design is the best method for a choice task design in real choice experiments. However, based on the attributes and their corresponding levels considered in this study, a total of 2^4^ × 4^1^ = 64 virtual choice profiles could be generated. Moreover, we used two choice profiles of pork, plus a “None of them” profile, totaling three alternative choices for each choice task. It is impractical for consumers to compare (64 × 63) ÷ 2 = 2016 groups of choice profiles, due to fatigue. According to Allenby and Rossi, fatigue will occur if participants identify more than 15–20 choice profiles [57,58]. Therefore, determining the number of choices sets and randomly designing the attribute combinations of pork using fractional factorial design (FFD) is essential for reducing biases and estimating all the cross-terms. To alleviate consumer fatigue, the number of choice tasks in each questionnaire was reduced to 9. Then, we used SSI Web 7.0 (Sawtooth Software, Provo, UT, U.S.A.) to design 10 different versions of the questionnaire to ensure that the questionnaire had the highest design efficiency. The verification results of the choice task design shows that the value of D-efficiency of all attributes is over 90.69%, and the frequency of all levels of all attributes in the experimental design was generally well balanced, and the bias relating to actual and ideal standard deviation was lower than 1%. A sample of the choice tasks is shown in Figure 2. No statistical methods were used to predetermine the sample sizes, but our sample sizes were similar to those reported in previous publications. For example, Francesc and Azucena [59] used a choice experiment to study the difference in the Valuation of Nutritional Claims among consumers, based on a sample of 121 Spanish respondents. De-Magistris and Lopéz-Galan [60] studied consumers’ WTP for nutritional claims, based on a sample of 219 consumers. Denver et al. [33] studied consumer preferences relating to pig welfare, based on an online questionnaire, with a choice experiment involving 396 Danish respondents. Based on these previous studies and our cost budget, we prepared data of 300–350 respondents and, finally, our sample consisted of 316 effective respondents. All subjects gave their informed consent for inclusion before they participated in the study. The study was conducted in accordance with the Declaration of Helsinki, and the protocol was approved by the Ethics Committee of Jiangnan University ([2018]118).

### 2.4. Experimental Organization and Implementation

#### 2.4.1. Experimental Area

Wuxi is located in the center of China’s Yangtze River Delta, and this city is one of the leading cities in terms of economic and social development in Jiangsu Province or even the whole country. In addition, Wuxi is a traceable pork pilot city, designated by the Ministry of Commerce of China. It should be noted that if a project regarding food traceability is conducted in a city where traceable food has not been piloted, researchers have to explain related concepts in detail as the consumers may not understand the meaning of traceable food. This would not only greatly increase the time needed to collect data, but it may also introduce bias, as the survey results may be dependent on the explanation of the concepts (i.e., whether our explanation is perceived to be neutral by all participants) and how the explanation is conducted during the surveys (e.g., in person or on paper). In order to mitigate these potential problems, the data in this paper were collected in a city where traceable food had been introduced as a pilot project for several years. In this study, data were randomly collected in seven administrative regions of Wuxi (including Chongan, Nanchang, Beitang, Binhu, Xishan, Huishan, and Wuxi New District) to increase the representativeness of the sample. Nowadays, many Chinese consumers like to buy food in supermarkets. Pork with quality and safety certification attributes, grain-fed pork, etc., are generally sold in large supermarkets. Thus, experimenters from the Jiangsu Province Food Safety Research Institute randomly intercepted consumers near the front gates of large supermarkets in the seven administrative regions. At each supermarket, the experimenters chose the third consumer coming into their sight. A filter question concerning the frequency of household food purchasing was used to screen participants at the beginning of the interview. Participants who answered that they are responsible for less than half of their household food purchase were excluded from the study. There were 46 participants selected from each administrative region, and they were divided into two groups, with each group consisting of 20–24 participants. Each experiment with each group lasted about 1 h. A total of 322 participants were recruited. The same group of participants completed the same version of the questionnaire. There were 10 versions of the questionnaire and 14 groups of participants in total. Four versions of the questionnaire were randomly selected for the remaining four groups. Ultimately, all 90 (9 × 10) groups of pork choice profiles generated from the 10 versions of the questionnaire were included in the experiment. All experiments were completed from September 2nd to September 11th, 2018.

#### 2.4.2. The Experimental Steps and Process

Before the experiment, the experimenter placed 9 transparent glass boxes on a table at the same time, each corresponding to the choice task cards (1st to 9th) in the questionnaires, and each box contained two pieces of pork corresponding to options A and B in the task (Figure 1). A traceability label, which displayed a traceable code, a lean meat essence detection information label, an animal welfare label, and the price, was posted on each pork package. The experiment was initiated when all of the participants had arrived. The specific steps of the experiment were as follows:

(a) Step 1: After recruiting a group of participants, the experimenters gave each participant 20 yuan and an ID number. Participants sit in the appropriate seats in accordance with their respective ID numbers. Participants were not allowed to discuss with each other. Participants were informed in advance that they needed to actually pay for the pork with the compensation they received.
(b) Step 2: Participants were informed of the experimental objective, experimental process, and questionnaire details, and they were informed that the pork presented to them had different traceability information, lean meat essence levels, animal welfare and freshness, but no differences in the brand.
(c) Step 3: When the experiment began, the participants were reminded to observe the pork in the box and were able to select the appropriate options in the questionnaire in any order.
(d) Step 4: At the end of the experiment, one task card was selected randomly from 9 task cards. All participants actually paid for the option they chose from the binding choice set, and they received the corresponding pork hindquarters. Taking Figure 2 as an example, if a consumer chose option A, he/she would need to pay 16 yuan and would receive 500 g of pork hindquarters with traceability code, fresh appearance, and no clenbuterol. Participants do not have to pay and buy any pork if they chose the ‘None of them’ option. Because of the randomness of the final real choices made by the participants, it would cost too much to prepare all of the pork products according to all of the possible results. Therefore, the following method was actually used. We bought the pig hindquarters that could be traced, had detected no lean meat essence, and a stocking density that met the national standard, and prepared four kinds of attribute labels, namely traceable code, detected no lean meat essence, meets the national stocking density standard, and price. The number of pieces of pork, prepared for use in the real exchange, was equal to the number of participants. Finally, each piece of pork was posted with a corresponding attribute label, according to the binding choice set randomly selected by the participant, and then provided to the participant (in order not to affect the whole research process and results, this process was confidential to participants). When the pork selected by consumers in the binding choice set was labeled with “detected no lean meat essence”, which displays the test result that the product did not contain lean meat essence, otherwise this pork product did not have a label indicating the lean meat essence test result.

In addition, participants needed to complete other parts of the questionnaire after the experiment, including their individual characteristics and perception of each attribute. A total of 322 consumers were randomly recruited, 316 of whom participated in the experiments; the others were not included in this experiment because they failed to fill in the questionnaires. The experimental procedure of our RCE closely followed that of Azucena et al. [51] and Chen et al. [61].

## 3. Results

### 3.1. Respondents’ Individual Characteristics and Consumption and Perception of Pork

As shown in Table 2, the majority of the 316 respondents in this survey were women, accounting for 52.22%, which is consistent with the fact that women are the major food buyers in urban Chinese households. In total, 17.40% of the participants were aged under 25 years; 31.33% of the participants were aged 26–35 years; 15.19% of the participants were aged 36–45 years; and 12.98% of the participants were aged over 56 years. In addition, 22.15% had a junior college education and 23.42% had an undergraduate-level education. As for the annual household income, 18.36% and 11.39% of the respondents’ annual household income ranged 111,000–150,000 and 151,000–200,000 RMB (China Dollars), respectively, and 14.87% earned more than 200,000 RMB. Importantly, according to the Wuxi and National Bureau of Statistics Survey Office, in 2017, 49.29% and 50.71% of the urban population of Wuxi were male and female, respectively, and 10.30%, 65.22%, and 24.48% were aged less than 15 years, 15–59 years, and more than 60 years, respectively. The demographic characteristics of the samples in this paper did not match the demographic characteristics of Wuxi very well.

In the case of pork consumption, the weekly pork consumption of the respondents was concentrated at “0.5–1 kg” and “1.1–1.5 kg”, accounting for 31.65% and 24.68%, respectively. In this study, the main places for purchasing pork were farmers’ markets and supermarkets. Of the respondents, 59.49% valued “freshness” as the most important characteristic when purchasing pork, and 32.28% of consumers paid the most attention to the “quality” of pork. Up to 68% of the respondents thought that the food safety levels of Chinese pork were “normal” or “unsafe”.

Table 3 shows the respondents’ perceptions and attitudes concerning the lean meat essence incident, the pork traceability system, and animal welfare. Of the 316 respondents, 84.81% were aware of the lean meat essence incident, and 49.68% and 38.61% considered lean meat essence tests on pork and its relevant products “very necessary” and “necessary”, respectively. About 61.08% of the respondents had relatively lower levels of understanding of the pork traceability system (i.e., below average or no understanding), and 26.58% had an average understanding. However, just 2 respondents had an excellent understanding of the pork traceability system, and 31.65% were not sure. In this survey, up to 66.77% of the respondents had rather lower levels of understanding of animal welfare (i.e., below average or no understanding). In addition, 68.35%, 11.39%, 17.41%, 2.53%, and 0.32% of the respondents believed that improving animal welfare is “extremely helpful”, “helpful to some extent”, “unsure”, “not helpful”, and “useless” for improving pork quality and safety, respectively.

### 3.2. Estimated Consumer Preferences by the RPL and LCM Models

#### 3.2.1. Model Selection and Construction

The data collected were analyzed assuming that in each choice task respondents behave according to random utility models [2].

According to the random utility theory, it is possible to divide utility into a deterministic and a stochastic part. If *U_nit_* is the utility obtained by consumer *n* participating in the experiment and choosing pork hindquarters *i* in choice task C in situation t, utility *U_nit_* includes two parts: the deterministic term *V_nit_* and the stochastic term *ε_nit_*, i.e.,

(1) $$U_{nit}=V_{nit}+\varepsilon_{nit}$$

Therefore, consumer *n* will choose alternative *i,* if $U_{nit}>U_{njt}.\forall j\neq i$. Consequently, the probability of consumer *n* choosing alternative *i* is given by

(2) $$P_{nit}=prob(V_{nit}-V_{njt}>\varepsilon_{njt}-\varepsilon_{nit};\forall j\neq i)$$

*V_nit_* is a linear function of the attributes of the five pork hindquarters, including traceability, lean meat essence test, animal welfare, appearance, and price:

(3) $$V_{nit}=\beta_{n}^{\prime }X_{nit}$$

where $\beta_{n}^{\prime }$ is a vector of the random parameters, which has its own mean and variance representing the individual preferences, and *x_nit_* is the vector of attributes including the profitability factor found in the *i*th alternative.

Because consumer preferences are assumed to be heterogeneous, this study examines a random effects specification by implementing a random parameter logit (RPL) model [62,63], and in our models, all random parameters were assumed to be normally distributed. If it is assumed that *ε_nit_* follows a type I maximum extreme value distribution, the probability of consumer *n* choosing pork hindquarters *i* under condition t can be expressed as

(4) $$P_{nit}=\int \frac{\exp(V_{nit})}{\sum j\exp(V_{niit})}f\left(\beta_{n}\right)d\beta_{n}$$

where *f*(β) is the probability density function of parameter β. If *f*(β) is discrete, then Equation (4) can be converted into a latent class model (LCM). In the LCM, *f*(β) takes S to be a distinct value. The probability that consumer *n* selects option *i* in a given choice situation t, irrespective of the class, is expressed as

(5) $$P_{nit}=\sum_{s=1}^{S}\frac{\exp(\beta_{s}X_{nit})}{\sum j\exp(\beta_{s}X_{njt})}R_{ns}$$

where $\beta_{s}$ is the parameter vector of the consumer group in class *s* and $R_{ns}$ is the probability of consumer *n* falling into class *s*. The corresponding probability can be expressed as

(6) $$R_{ns}=\frac{\exp(\theta_{s}{}^{\prime }Z_{n})}{\sum r\exp(\theta_{r}{}^{\prime }Z_{n})}$$

where $Z_{n}$ is a range of observed values influencing consumer *n* in a certain class, and ${\theta_{s}}^{\prime }$ is the parameter vector of consumers in class *s*.

The attributes and levels in Table 1 were coded using dummy coding, while the price was a continuous variable. “None of them” was coded as a dummy variable, which takes a value of 1 for the non-buying option and a value of 0 otherwise. Consumers’ WTP for each attribute level can be estimated using consumers’ part-worth utilities for each attribute level and price, calculated by the RPL and LCM models [63]. Because the variables were dummy coded in this paper, according to Francesc [59] and De-Magistris and Lopéz-Galan [60], the WTP can be represented as

(7) $$\mathrm{WTP}=-\frac{\beta x}{\beta p}$$

Interaction WTP=βx+βm×INTERACTION where βx and βp represent the x-attribute coefficient and price coefficient, respectively; m donates the interaction terms, INTERACTION is product characteristic (for example, dummy = 1 if pork product labeled “traceable code” and “Detected no lean meat essence”).

#### 3.2.2. Consumer Preferences Relating to Different Attributes and Their Classification

According to Ubilava and Foster, we assume that the coefficients of the “None of them” variable, price, and interaction terms were fixed, and that the parameters of the other attributes were randomly and normally distributed [64]. By calculating the ratio of choosing the “None of them” option, we found that there were 76 people who selected “None of them” once or more in their experiment questionnaire, accounting for 24.05%. Among the total of 2844 (316 × 9) observations in the whole experiment, 251 observations selected “None of them”, accounting for 8.826%. The utility value of the different attributes was calculated using NLOGIT 5.0. (manufactured by ECONOMETRIC Software Inc., Plainview, NY, USA) The regression result of the RPL model is shown in Table 4. The price attribute was significant at 1%, and the coefficient was negative. The utility values of traceable code, detected no lean meat essence, a national stocking density standard of animal welfare, and bright red appearance attributes were positive and significant at 1%, indicating that consumers attached a high importance to these attributes. According to the method and formula, suggested by Troiano et al. [65,66], the relative importance of traceability, appearance, animal welfare, lean meat essence test, and price was calculated and found to be 15%, 11%, 9%, 8%, and 57%, respectively.

The importance of price ranks as number one. Lagerkvist [67] and Merlino et al. [20] also found the price consideration to be the most important characteristic of meat, compared with, for example, the origin or nutritional aspects of meat. Curtis and Dolling [68] found consumers considered the price as an “extremely important” attribute relating to meat choice.

Traceability ranked as the second most important attribute. This conclusion is similar with the research result from Stranieri et al. [66]. In other words, traceability is an important attribute relating to food quality and safety, despite the fact that Lagerkvist [67] and Merlino et al. [20] reported that information about traceability was not perceived as an important quality cue by consumers. This paper showed that consumers prefer pork products with traceability because it represents pork safety during the whole process of pork production, including farming information (e.g., breeding time, swine supplier, quarantine), slaughtering information (e.g., pre-slaughter quarantine, pork quarantine, and slaughtering time), and transport and retail information (e.g., transport time, mode, and retailer), rather than attributes that only narrowly represent pork quality, such as animal welfare and lean meat essence testing. This conclusion was also sustained by research from Abidoye [25], Plessis and Rand [26], and Wu et al. [18]. In China, most pork safety issues occur during the swine breeding period [18]. Therefore, although lean meat essence testing information is more intuitive, overall, consumers expect to receive complete processing quality and safety information, from farming to slaughter and transport.

The results of this paper indicate that a bright red appearance comes next in importance, after price and traceability, and it is more important than a national stocking density standard of animal welfare and detected no lean meat essence. Many other researches also indicated that appearance was one of the attributes that consumers are concerned with when purchasing meat products. In the United States and Argentina, consumers were willing to pay more for beef with a fresh red color [44]. Berges et al. [45] and Merlino et al. [20] believed that the color of meat played a remarkable role during purchasing for interviewees who consumed a lot of meat during the week and regularly shopped at supermarkets. Chinese consumers prefer to purchase bright red pork [27], as the color of the meat usually reflects its freshness.

Merlino et al. [20] concluded that animal welfare is the second most important attribute considered during meat purchasing by Piedmontese consumers. Recent studies focusing on the European Union indicated that consumers are willing to eat animal-friendly meat because they associate it with a higher quality and health benefits [69]. Torquati et al. [70] found that consumers in the European Union pay significant attention to animal welfare, and their WTP for a pack of 6 organic eggs, produced in compliance with animal welfare standards, was €2.16, a value 3 times higher than the value estimated for the “social work” attribute and 4 times higher than the value estimated for the “local” attribute. However, in Miele [71], differences in the importance that consumers attached to animal welfare emerged in comparative works, including southern and Scandinavian European countries (seven in total), finding that of all of the studied countries, French and Dutch consumers were the least interested in animal welfare, the British were at the center, while Hungarian, Swedish, Norwegian, and Italian consumers were the most interested in the animal welfare issue. Generally, compared to Chinese consumers, other countries’ consumers pay more attention to the animal welfare attribute. This paper reveals that the importance of animal welfare information (stocking density meet the national standard) follows price, traceability code and bright red appearance for Chinese consumers, but it is higher than detected no lean meat essence. Another study focusing on Chinese consumers, by Lai et al. [34], indicated that among the food safety, origin country, environmental labeling, animal welfare, and price attributes, Chinese consumers’ preferences relating to animal welfare came after food safety, origin country (China), and environmental labeling, but was prior to the origin country (the United States) and price, which was similar to the result of this paper. Therefore, Chinese consumers have a potential demand for the animal welfare attribute, and they may start to realize the interrelationships of meat safety, taste, and animal welfare.

In this paper, consumers were found to have a potential, but not high, demand for detected no lean meat essence. Apart from the traceability and price attributes, the preference of detected no lean meat essence comes slightly after the bright red appearance of meat and a national stocking density standard of animal welfare. On the contrary, there were some papers that indicated that consumers had a very high preference for growth hormones in meat products [5,39]. This is likely because this paper did not investigate growth hormones, which were replaced by lean meat essence in the national conditions of China. Another possibility is that there were diverse demands and preferences for lean meat essence among different type of consumers, some of which paid significant attention to and had a preference for detected no lean meat essence, which will be discussed further in a subsequent section of this paper on consumer classification and the WTP of pork attributes.

The estimated models also include interaction variables that capture any interaction effects between main-effect attributes. The results show that the interaction terms of traceable code and bright red appearance, detected no lean meat essence, and a national stocking density standard of animal welfare are both significant at 10% and positive, respectively, indicating that traceability and appearance, lean meat essence test, and animal welfare are complementary. This may be because a bright red color and traceable pork make consumers feel safe, whereas the lean meat essence testing attribute, since it can only detect the existence or inexistence of lean meat essence, cannot reflect whether the swine breeding density meets the national criterion or whether the breeding environment is excellent, unlike the animal welfare attribute.

A further analysis of preference heterogeneity was conducted to search for the presence of potential clusters of respondents with homogeneous preferences. In the RPL model, the variances in traceable code, bright red appearance, detected no lean meat essence, and a national stocking density standard of animal welfare are significant at 1%, 1%, 5%, and 10%, respectively, indicating consumer preference variances. Therefore, the LCM model was applied for further analysis. To determine the best number of classes, we estimated the model using two, three and four latent classes and calculated four information criteria: the Akaike information criterion (AIC), the modified Akaike information criterion (AIC3), the Bayesian information criterion (BIC), and tρ^2^, also called the Akaike likelihood ratio index. The preferred model should be the one with the lowest AIC, AIC3, and BIC, and the highest ρ^2^ [72]. The calculated information criteria were constantly decreasing or increasing. However, the improvement from two to three segments was greater than the change from three to four (Table 4). In the model for three classes, the values of AIC and AIC3 were the lowest, and the value of ρ^2^ was the highest. Moreover, we observed in the model for four classes that the value of the estimated parameters started to deteriorate, giving a larger AIC, AIC3 and BIC, which is considered as an indication to stop looking for more classes. Thus, consumers were classified into three categories, according to their gender, age, education, income, marriage and children, as covariates based on the optimal category choice.

As shown in Table 5, the utility value of traceable code, detected no lean meat essence, a national stocking density standard of animal welfare, and bright red appearance are significant for consumers in Class 1, which are called “quality-focused” consumers, accounting for 56.9% of the sample. This reveals that over half of the consumers in this research care about the pork quality attribute. Meanwhile, compared with traceable code and detected no lean meat essence, a national stocking density standard of animal welfare is only significant at the 10% level with the lowest coefficient, indicating there is potential cognitive promotion in animal welfare for Chinese consumers, especially in the interrelationship of meat quality and safety, premium animal welfare, and processing measures. This conclusion was also supported by the research of Lewis et al. [5]. For Class 2 consumers, the price is significant at 1%, while the other attributes show no significance; therefore, this type of consumer is called a “price-sensitive” consumer, accounting for 28.1% of the sample. In terms of the covariates, age is significant at 10% with a positive coefficient, indicating that older consumers are more sensitive to price. Marriage is significant at 10%, with a negative coefficient, indicating that spinsterhood and divorced or widowed consumers are also sensitive to price. For Class 3 consumers, the price is significant at 1%, and the true value of the coefficient is between Class 1 and Class 2, accounting for 15% of the sample. Besides being sensitive to price, the interaction terms of traceability and detected no lean meat essence, traceability, and bright red appearance are both significant at 10%, with a positive coefficient, indicating that there is complementary relationship between traceability and lean meat essence testing or appearance. In other word, Class 3 consumers believe that pork with a traceability code and detected no lean meat essence, or pork with a traceability code and bright red appearance, are safer. Therefore, this type of consumer is called a “preference combination” consumer.

#### 3.2.3. Consumers’ WTP Relating to Attribute Levels

As shown in Table 6, in the RPL model, the average consumer WTP for traceable code is 4.76 yuan/catty, which is lower than the result found by Wu et al. [73], who found that consumers are willing to pay an extra 8.32 yuan/catty for traceable pork, which not only contains complete information on all of the processing stages, but also has government certification.

The results of this paper suggest that consumers have a potential demand for detected no lean meat essence, a national stocking density standard of animal welfare, and bright red appearance attributes, with a WTP of 2.36, 2.86, and 3.36 yuan/catty, respectively, was averaging more than 2 yuan/catty. Lai et al. [34] investigated the willingness of a Jiangsu consumer to pay a premium for animal welfare and found that the premium price was 2.56 yuan/catty when animal welfare and meat quality information were provided, which is a little lower than the results found in this paper. Lai et al. [34] reported that the premium price that consumers would like to pay for animal welfare was 7.65 and 13.11 yuan/catty in Beijing and Shanghai, respectively, without risk cognition, both of which are higher than the result of 2.86 yuan/catty, found in this paper. The main reason is economic variations in the different investigated cities. In 2018, the per capita disposable income (PCDI) of Beijing, Shanghai, Wuxi, and Jiangsu was 62,361, 64,183, 50,373, and 38,096 RMB, respectively. Wuxi has a higher level of economic development than the average of Jiangsu province, but it is still lower compared with Shanghai and Beijing. As for the interaction attributes, consumers show a preference for the interaction terms of traceable code combined with a bright red appearance, as well as detected no lean meat essence combined with a national stocking density standard of animal welfare, and they are willing to pay 1.79 and 2.24 yuan/catty, respectively. Consumers are also willing to pay 1.89 and 1.04 yuan/catty for interaction attributes of traceable code and detected no lean meat essence, as well as detected no lean meat essence and a bright red appearance, respectively.

There are vast variations of WTP from the perspective of consumer classification, according to the LCM model cluster membership. In the LCM model, “quality-oriented” consumers have the highest WTP for each single attribute, especially for traceable code, which is as high as 20.65 yuan/catty. Their premium price for detected no lean meat essence is 16.38 yuan/catty; for a bright red appearance, it is 14.69 yuan/catty; and for a national stocking density standard of animal welfare, it is 10.40 yuan/catty, which is the lowest. Compared with the other two types of consumers, “quality-oriented” consumers value the lean meat essence test more highly, and the importance is higher than that of animal welfare and appearance. In terms of interaction attributes, they only prefer traceable code combined with a bright red appearance, as well as detected no lean meat essence combined with a national stocking density standard of animal welfare, and they are willing to pay 3.76 and 3.84 yuan/catty, respectively. An opposing example is “price-sensitive” consumers, who show an extremely low WTP for each single attribute. Their WTP for a traceable code, a national stocking density standard of animal welfare, and a bright red appearance are below 1.5 yuan/catty. Their WTP for the interaction attributes is also very low. This type of consumer refers mainly to elder, unmarried, divorced, or widowed consumers. “Preference combination” consumers show variances for different attributes. Their WTP for a traceable code and a national stocking density standard of animal welfare is about 4.79 and 5.37 yuan/catty, respectively; for detected no lean meat essence and a bright red appearance, the values are both negative. Overall, this type of consumer values interaction attributes more highly than the other two types of consumers. Especially when traceable code is combined with detected no lean meat essence or a bright red appearance and detected no lean meat essence is combined with a national stocking density standard of animal welfare or a bright red appearance, “preference combination” consumers tend to be more trusting. Among these interaction attributes, their WTP for traceable code combined with a bright red appearance is the highest, at 6.28 yuan/catty.

## 4. Discussion and Conclusions

In this paper, as a case of consumer preference, 316 consumers in Wuxi, China, were investigated in terms of their preferences in relation to pork attributes, such as traceability, lean meat essence test, animal welfare, appearance, and price. The preferences and WTP for the attributes were assessed using a choice experiment, followed by real purchases. The main conclusions are as follows:

(a) Of the respondents, 59.49% valued “freshness” as the most important characteristic when purchasing pork, and 32.28% of the respondents paid the most attention to the “quality” of pork. Up to 68% of the respondents thought that the food safety levels of Chinese pork are “normal” or “unsafe.” The respondents had a high awareness of lean meat essence incidence, but lower levels of understanding of the pork traceability system and animal welfare. Approximately 80% of the respondents thought that it is necessary to conduct lean meat essence tests and improve animal welfare in the pork industry.
(b) The consumer pays the most attention to the price attribute, followed by the traceability, appearance, and animal welfare, and the least important attribute is the lean meat essence test. The consumer’s WTP of the traceable code is 4.76 yuan. The WTP of the bright red appearance, a national stocking density standard of animal welfare, and the detected no lean meat essence attribute is above 2 yuan. Since the consumers attach different levels of importance to the pork properties, they can be divided into “quality-focused”, “price-sensitive”, and “preference combination” consumers, accounting for 56.9%, 28.1%, and 15% of the sample, respectively.
(c) For consumers, there is a complementary interrelationship between the traceability and appearance attributes, lean meat essence test, and animal welfare attributes. On the one hand, pork has a comprehensive traceability and bright red appearance, ensuring the quality and safety of pork. On the other hand, the lean meat essence test attribute can only check whether or not illegal additives have been used in pigs’ feed, but it cannot guarantee that pigs’ breeding density meets the national standards, nor whether it is abused, whether the breeding environment is comfortable, etc., whereas animal welfare attributes can provide such information.
(d) “Quality-focused” consumers pay more attention to the quality and safety of pork, and they are willing to pay a high price for traceability information, lean meat essence testing information, animal welfare information, and appearance attributes. Their highest WTP for the traceability attribute could be partially driven by the incidence of African swine fever virus (ASFV) in August 2018. The experiment was conducted immediately after that incident. Consumers’ excessive exposure to mass media reports on ASFV may have made participants pay more attention to the traceability attribute. Thus, our research group will conduct follow-up surveys to check the informational bias effects. “Price-sensitive” consumers pay significant attention to the price, and the price paid for each product is meagre. Such consumers are usually older and are mostly unmarried, divorced, or widowed. “Preference combination” consumers are less sensitive to the price than “price-sensitive” consumers but are more sensitive than “quality-focused” consumers. Compared to the other two types of consumers, the “preference combination” consumers value interaction attributes more highly, like traceable code combined with detected no lean meat essence or bright red appearance, as well as detected no lean meat essence combined with a national stocking density standard of animal welfare or bright red appearance.

Based on the above conclusions, this paper provides the following reference values for the Chinese government and relevant production enterprises:

(a) While improving the traceability system, the government should increase the testing intensity, expand the scope of additives considered to be illegal (e.g., lean meat essence), and promote the welfare level of pigs in China. Additionally, the exposure of the lean meat essence testing information and animal welfare information should be given more attention to ensure food safety. Meanwhile, the government should promote public education and publicity on pork quality and safety attributes; this would provide consumers with a better understanding of traceability information, lean meat essence testing, animal welfare, and other attributes which, in turn, would guide consumers to develop a more comprehensive judgement of the issues surrounding the purchasing of pork, i.e., not only a product’s appearance attributes, but also other attributes (e.g., traceability or quality certification attributes).
(b) According to different consumer groups, enterprises can formulate “differentiated” pork products. For “quality-focused” consumers, companies can increase the testing and certification properties of pork and other relevant products (e.g., traceability properties, lean meat essence detection properties, or animal welfare properties), and the companies can appropriately increase prices, according to production costs, to meet the requirements for pork quality and safety for consumers. As for the “preference combination” consumers, enterprises can use a strategy combining the traceability attribute with a lower level of information and lean meat essence testing attribute, which would be helpful in controlling the price of high-quality pork and increasing consumers’ desire to purchase pork.

## 5. Limitations

(1) The results are biased because we considered a sample in Wuxi that is already familiar with the pork traceability system. We did not consider other consumers outside of the pilot area who are not familiar with the pork traceability system in China. In the future, we should compare our results with the behavior of consumers who are not familiar with the traceability system, to see whether there is a difference.
(2) We designed the level of the price attribute in the choice experiment according to the real price of normal pork on the Chinese market and previous studies. We acknowledge that the range of price levels (12, 14, 16, and 18) may be too small to reflect the cost incurred in attaining traceability, lean meat essence testing, and animal welfare certification. This may cause the WTP for the safety attributes to be particularly high for the “quality-oriented” group, not only for the traceability attribute but also for other attributes studied in this paper. This constitutes a research limitation of our study, and we should carefully think and design the price levels to adequately reflect the quality differentiation in the future.
(3) The third limitation of our paper is that it is indeed not clear how the level of knowledge regarding animal welfare or the traceability system was assessed in our paper. Rather, we allowed the respondents to evaluate this themselves.

## Figures and Tables

**Table 1:** The attributes of pork hindquarters and their corresponding levels.

Attributes	Levels	Explanation
Traceability	Traceable code displaying information on hog farming, slaughtering, processing, transportation, and sale	The traceable code provides hog farming information (including the slaughter time, producer, animal quarantine), slaughter process information (including the quarantine, before slaughtering, and pork quarantine), and transportation and sale information (including the transportation time, transportation method, and marketing
	No traceable code	Pork or other relevant products do not have a traceability code
Lean meat essence test	Detected no lean meat essence	Pork or other relevant products have a label displaying the test result that the product does not contain lean meat essence
	No information about lean meat essence	Pork or other relevant products do not have a label indicating the lean meat essence test result
Animal welfare	Meets the national stocking density standard	A label on the stocking density indicating that it meets the national standard, i.e., pigs’ weight is less than 20, 20–50, 50–80, 80–110, their corresponding minimum lying space is not less than 0.2, 0.4, 0.6, or 0.8 m^2^ per pig
	No information about animal welfare	No relevant label on pork or other relevant products
Appearance	Bright red	Bright red for very fresh
	Light red	Light red for generally fresh
Price	12 yuan/catty	The price of pork is 12, 14, 16, or 18 yuan/catty
	14 yuan/catty	
	16 yuan/catty	
	18 yuan/catty	

**Table 2:** Respondents’ individual characteristics.

Characteristics	Items	Sample Size	Percent (%)	The Census Data (%)
Gender	Male	151	47.78	49.29
	Female	165	52.22	50.71
Age	25 or younger	55	17.40	10.30 ^a^
	26–35	99	31.33	65.22 ^a^
	36–45	48	15.19	
	46–55	41	12.98	
	Older than 56	73	23.10	24.48 ^a^
Education level	Middle school or lower	80	25.31	62.77
	High school	63	19.94	17.81
	Junior college	70	22.15	12.88 ^b^
	Undergraduate	74	23.42	
	Master or above	29	9.18	
Annual household income	70,000 yuan or less	95	30.06	*
	71,000–110,000 yuan	80	25.32	*
	111,000–150,000 yuan	58	18.36	*
	151,000–200,000 yuan	36	11.39	*
	200,000 yuan or above	47	14.87	*
Household weekly pork consumption	Less than 0.5 kg	38	12.03	*
	0.5–1 kg	100	31.65	*
	1.1–1.5 kg	78	24.68	*
	1.6–2 kg	41	12.97	*
	More than 2.1 kg	59	18.67	*
The most important characteristics of pork	Quality	102	32.28	*
	Price	15	4.75	*
	Freshness	188	59.49	*
	Brand	8	2.53	*
	Other	3	0.95	*

Note: ^a^ the age brackets used in the Wuxi population census are as follows: 0–14 years, 15–59 years, and more than 60 years; ^b^ represents the sum of the items; * indicates that the item is not included in the census result.

**Table 3:** The respondents’ cognition of lean meat essence, animal welfare, and traceability.

Characteristics	Items	Number of Samples	Percent (%)
The incident of lean meat essence (known or not)	Yes	268	84.81
	No	48	15.19
Necessity of a lean meat essence test on pork	Very necessary	157	49.68
	Necessary	122	38.61
	Dispensable	23	7.28
	Not necessary	11	3.48
	Not necessary at all	3	0.95
Levels of the understanding of the pork traceability system	Excellent	2	0.63
	Good	37	11.71
	Average	84	26.58
	Below average	146	46.20
	No understanding	47	14.88
Potential of the pork traceability system to decrease the risk of pork safety	Definitely	25	7.91
	Possibly	171	54.11
	Not sure	100	31.65
	Not significantly	15	4.75
	Not at all	5	1.58
Levels of understanding of animal welfare	Excellent	5	1.58
	Good	26	8.23
	Average	74	23.42
	Below average	164	51.90
	No understanding	47	14.87
Influence of the promotion of animal welfare on pork quality and safety	Extremely helpful	36	11.39
	Helpful to some extent	216	68.35
	Not sure	55	17.41
	Not helpful	8	2.53
	Useless	1	0.32

**Table 4:** Statistics to determine the optimal number of consumer segments.

Number of Segments	Number of Parameters (P)	Log-Likelihood (LL) at Convergence	AIC	AIC3	BIC	ρ^2^
2	25	−1853.22418	3756.44836	3781.44836	1952.63626	0.39865
3	38	−1816.42664	3708.85328	3746.85328	1967.53301	0.40627
4	51	−1805.91316	3713.82632	3764.82632	2008.71381	0.40547

Note: AIC (Akaike information criterion) is calculated using −2(LL − *p*); AIC3 (Bozdogan Akaike information criterion) is calculated using (−2LL + 3*p*); BIC (Bayesian information criterion) is calculated using (−LL + (*p* ÷ 2) × ln(*n*)); ρ^2^ is calculated using (1 – AIC ÷ 2 × restricted LL); restricted log-likelihood = −3123.35474.

**Table 5:** Parameter estimation results for the choice experiment using the random parameter logit (RPL) and latent class model (LCM) models.

Attribute	RPL Model	LCM Model		
		Class 1 (Quality Oriented)	Class 2 (Price Sensitive)	Class 3 (Preference Combination)
Price	−0.198 ***	–0.052 *	−0.621 ***	−0.207 ***
	(0.018)	(0.027)	(0.092)	(0.073)
Traceable code	0.943 ***	1.074 ***	0.538	0.991
	(0.170)	(0.254)	(0.507)	(0.757)
Detected no lean meat essence	0.468 ***	0.852 ***	−0.029	−0.365
	(0.168)	(0.246)	(0.609)	(0.693)
A national stocking density standard of animal welfare	0.567 ***	0.541 *	0.712	1.111
	(0.174)	(0.288)	(0.654)	(0.759)
Bright red appearance	0.695 ***	0.764 ***	0.862	−1.252
	(0.173)	(0.275)	(0.669)	(0.942)
Traceable code * Detected no lean meat essence	0.374	−0.062	0.510	0.973 *
	(0.199)	(0.233)	(0.575)	(0.586)
Traceable code * A national stocking density standard of animal welfare	0.130	−0.089	0.217	−0.546
	(0.198)	(0.293)	(0.538)	(0.930)
Traceable code * Bright red appearance	0.354 *	0.197	0.147	1.301 *
	(0.171)	(0.209)	(0.624)	(0.756)
Detected no lean meat essence * A national stocking density standard of animal welfare	0.443 *	0.201	−0.261	0.654
	(0.175)	(0.196)	(0.534)	(0.545)
Detected no lean meat essence * Bright red appearance	0.205	−0.014	0.215	1.138
	(0.176)	(0.253)	(0.473)	(0.796)
A national stocking density standard of animal welfare * Bright red appearance	0.081	−0.036	0.134	−0.080
	(0.182)	(0.286)	(0.606)	(0.735)
None of them	−3.857 ***	−2.117 ***	−11.489 ***	−2.658 ***
	(0.299)	(0.462)	(1.649)	(1.008)
STDEV (Traceable code)	0.673 ** (0.298)	–	–	–
STDEV (Detected no lean meat essence)	0.004 (0.351)	–	–	–
STDEV (A national stocking density standard of animal welfare)	0.054 (0.684)	–	–	–
STDEV (Bright red appearance)	1.050 *** (0.214)	–	–	–
STDEV (Traceable code * Detected no lean meat essence)	1.109 *** (0.330)	–	–	–
STDEV (Traceable code * A national stocking density standard of animal welfare)	1.119 *** (0.339)	–	–	–
STDEV (Traceable code * Bright red appearance)	0.030 (0.327)	–	–	–
STDEV (Detected no lean meat essence * A national stocking density standard of animal welfare)	1.027 *** (0.373)	–	–	–
STDEV (Detected no lean meat essence * Bright red appearance)	0.067 (0.482)	–	–	–
STDEV (A national stocking density standard of animal welfare * Bright red appearance)	0.005 (0.288)	–	–	–
Age	NA	0.063	0.067 *	–
	NA	(0.038)	(0.041)	–
Edu	NA	−0.055	−0.066	–
	NA	(0.095)	(0.099	–
Income	NA	0.012	0.025	–
	NA	(0.051)	(0.057)	–
	NA	−0.834	−2.064 *	–
Married	NA	(1.066)	(1.089)	–
Male	NA	0.505	0.878	–
	NA	(0.723)	(0.747)	–
A child under 18 years in the family	NA	0.313	0.877	–
	NA	(0.805)	(0.850)	–
Class Prob.	NA	0.569	0.281	0.150
Number of observations	2844	1618	799	427
Pseudo R-squared	0.391	0.417		
Log-likelihood	−1900.041	−1820.308		

Note: ***, **, and * denote significance at the 1%, 5%, and 10% significant levels, respectively. Numbers in brackets refer to the standard deviation of the variable. Age and Edu were used as continuous variables, in which Edu referred to education level and measured by years of schooling. Income variable was income classes. STDDEV—Standard deviation; RPL—random parameter logit; LCM—latent class model.

**Table 6:** Consumer willingness to pay (WTP) for each attribute, estimated by the RPL and LCM models (yuan/500 g).

Attribute	Average WTP	Class 1 (Quality Oriented)	Class 2 (Price Sensitive)	Class 3 (Preference Combination)
Traceable code	4.76 (0.825)	20.65 (2.291)	0.87 (0.129)	4.79 (1.611)
Detected no lean meat essence	2.36 (0.500)	16.38 (2.008)	–0.05 (0.084)	−1.76 (1.576)
A national stocking density standard of animal welfare	2.86 (0.708)	10.40 (1.274)	1.15 (0.056)	5.37 (0.994)
Bright red appearance	3.36 (1.839)	14.69 (2.351)	1.39 (0.060)	−6.05 (1.407)
Traceable code Detected no lean meat essence	1.89 (1.484)	–1.18 (1.078)	0.82 (0.008)	4.70 (1.295)
Traceable code A national stocking density standard of animal welfare	0.66 (1.566)	−1.69 (0.180)	0.35 (0.018)	−2.64 (0.345)
Traceable code—Bright red appearance	1.79 (0.802)	3.76 (0.542)	0.24 (0.033)	6.28 (1.420)
Detected no lean meat essence—A national stocking density standard of animal welfare	2.24 (1.237)	3.84 (0.302)	–0.42 (0.036)	3.16 (0.748)
Detected no lean meat essence—Bright red appearance	1.04 (0.607)	−0.27 (1.196)	0.35 (0.027)	5.49 (1.338)
A national stocking density standard of animal welfare—Bright red appearance	0.41 (0.710)	−0.69 (0.051)	0.22 (0.010)	−0.39 (0.223)

Note: Standard errors are in parenthesis. WTP—willingness to pay; RPL—random parameter logit; LCM—latent class model.

## References

[1] Aung M.M., Chang Y.S. (2014). Traceability in a food supply chain: Safety and quality perspectives. Food Control.

[2] Lancaster K.J. (1966). A new approach to consumer theory. J. Political Econ..

[3] Hobbs J.E., Bailey D.V., Dickinson D.L., Haghiri M. (2005). Traceability in the Canadian Red Meat Sector: Do Consumers Care?. Can. J. Agric. Econ..

[4] Samant S.S., Seo H.S. (2016). Quality perception and acceptability of chicken breast meat labeled with sustainability claims vary as a function of consumers’ label-understanding level. Food Qual. Prefer..

[5] Lewis K.E., Grebitus C., Colson G., Hu W. (2017). German and British consumer willingness to pay for beef labeled with food safety attributes. J. Agric. Econ..

[6] Steenkamp J.B.E.M. (1990). Conceptual model of the quality perception process. J. Bus. Res..

[7] CalvoDopico D., Mendes R., Silva H.A., Verrez-Bagnis V., Pérez-Martín R., Sotelo C.G. (2016). Evaluation, signalling and willingness to pay for traceability. A cross-national comparison. Span. J. Mark.-ESIC.

[8] Wang J.H., Gao Z.Q., Shen M.M. (2018). Recognition of Consumers’ Characteristics of Purchasing Farm Produce with Safety Certificates and Their Influencing Factors. Int. J. Environ. Res. Public Health.

[9] Shen J.Z., Jiang H.Y. (2011). Residues of β-adrenergic agonist in Animal Products and Its Hazards. Chin. J. Anim. Health. Insp..

[10] Blanca J., Muñoz P., Morgado M., Reuvers T., Nely M., Aranda A., Hooghuis H. (2005). Determination of clenbuterol, ractopamine and zilpaterol in liver and urine by liquid chromatography tandem mass spectrometry. Anal. Chim. Acta.

[11] Hieger M.A., Emswiler M.P., Maskell K.F., Sentz J.T., Miller K.B., Wolf C.E., Cumpston K.L., Wills B.K. (2016). A case series of clenbuterol toxicity caused by adulterated heroin. J. Emerg. Med..

[12] Fan Y.H. (2016). Regulation and Countermeasure of “Leptin”.

[13] Hartung J., Nowak B., Springorum A.C. (2009). Animal Welfare and Meat Quality.

[14] Velarde A., Fabrega E., Blanco-Penedo I., Dalmau A. (2015). Animal welfare towards sustainability in pork meat production. J. Meat Sci..

[15] Gregory N.G., Grandin T. (1998). Animal Welfare and Meat Science.

[16] Harper G.C., Henson S.J. (1999). The Nature of Consumer Concerns about Animal Welfare.

[17] Wang J.H., Deng Y.Y., Diao H.Y. (2018). Perceived risk, expected benefits and pig farmers’ behaviors of veterinary drug usage. Int. J. Environ. Res. Public Health.

[18] Wu L.H., Gong X.R., Qin S.S., Chen X.J., Zhu D., Hu W. (2017). Consumer preferences for pork attributes related to traceability, information certification, and origin labeling: Based on China’s Jiangsu Province. Agribusiness.

[19] Lagerkvist C.J., Berthelsen T., Sundström K., Johansson H. (2014). Country of origin or EU/non-EU labelling of beef? Comparing structural reliability and validity of discrete choice experiments for measurement of consumer preferences for origin and extrinsic quality cues. Food Qual. Prefer..

[20] Merlino V.M., Borra D., Girgenti V., DalVecchio V.A., Massaglia S. (2018). Beef meat preferences of consumers from Northwest Italy: Analysis of choice attributes. J. Meat Sci..

[21] Sonoda Y., Oishi K., Chomei Y., Hirooka H. (2018). How do human values influence the beef preferences of consumer segments regarding animal welfare and environmentally friendly production?. J. Meat Sci..

[22] Troy D.J., Kerry J.P. (2010). Consumer perception and the role of science in the meat industry. J. Meat Sci..

[23] Dickinson D.L., Bailey D.V. (2005). Experimental Evidence on Willingness to Pay for Red Meat Traceability in the United States, Canada, the United Kingdom, and Japan?. J. Agric. Appl. Econ..

[24] Lu J., Wu L., Wang S., Xu L. (2016). Consumer preference and demand for traceable food attributes. Br. Food J..

[25] Abidoye B.O., Bulut H., Lawrence J.D., Bulut H., Townsend A.M. (2011). US Consumers’ Valuation of Quality Attributes in Beef Products. J. Agric. Appl. Econ..

[26] Du Plessis H.J., DuRand G.E. (2012). The significance of traceability in consumer decision making towards Karoo lamb. Food Res. Int..

[27] Wu L.H., Wang H.S., Zhu D. (2016). Chinese consumers’ willingness to pay for pork traceability information-the case of Wuxi. Agric. Econ..

[28] Chen X.J., Qin S.S., Yin SH.J., Wu L.H. (2016). Research on traceable pork supply side reform based on consumers’ preference for origin attribute information. China Popul. Res. Environ..

[29] Contini C., Romano C., Boncinelli F., Scozzafava G., Casini L. (2017). Does ‘local’ matter in restaurant choice? Results of a discrete choice experiment targeting German and Italian consumers. Agric. Food Econ..

[30] Li X.G., Jensen K.L., Lambert D.M., Clark C.D. (2018). Consequentiality Beliefs and Consumer Valuation of Extrinsic Attributes in Beef. J. Agric. Appl. Econ..

[31] Miranda-delaLama G.C., Estévez-Moreno L.X., Sepúlveda W.S., Estrada-Chavero M.C., Rayas-Amor A.A., Villarroel M., María G.A. (2017). Mexican consumers’ perceptions and attitudes towards farm animal welfare and willingness to pay for welfare friendly meat products. J. Meat Sci..

[32] Clark B., Stewart G.B., Panzone L.A., Kyriazakis I., Frewer L.J. (2017). Citizens, consumers and farm animal welfare: A meta-analysis of willingness-to-pay studies. Food Policy.

[33] Denver S., Sandoe P., Christensen T. (2017). Consumer preferences for pig welfare-Can the market accommodate more than one level of welfare pork?. J. Meat Sci..

[34] Lai J., Wang H.H., Ortega D.L., Widmar N.J.O. (2018). Factoring Chinese consumers’ risk perceptions into their willingness to pay for pork safety, environmental stewardship, and animal welfare. Food Control.

[35] Wang C.W., Gu H.Y. (2016). Animal welfare cognition and food safety of residents. J. Financ. Econ..

[36] Spain C.V., Freund D., Mohan-Gibbons H., Meadow R.G., Beacham L. (2018). Are they buying it? United states consumers’ changing attitudes toward more humanely raised meat, eggs, and dairy. Animals.

[37] Frey U.J., Pirscher F. (2018). Willingness to pay and moral stance: The case of farm animal welfare in Germany. PLoS ONE.

[38] Lusk J.L., Roosen J., Fox J.A. (2003). Demand for Beef from Cattle Administered Growth Hormones or Fed Genetically Modified Corn: A Comparison of Consumers in France, Germany, the United Kingdom, and the United States. Am. J. Agric. Econ..

[39] Yang S.H., Monteiro D.S., Chan M.Y., Woods D.A. (2016). Preferences for Meat Labeling in Taiwanese Traditional Markets: What do Consumers Want?. J. Food Distrib. Res..

[40] Viegas I., Nunes L.C., Madureira L., Fontes M.A., Santos J.L. (2014). Beef credence attributes: Implications of substitution effects on consumers’ WTP. J. Agric. Econ..

[41] Ellison B., Brooks K., Mieno T. (2017). Which livestock production claims matter most to consumers?. Agric. Hum. Values.

[42] Merritt M.G., DeLong K.L., Griffith A.P., Jensen K.L. (2018). Tennessee Consumers’ Willingness to Pay for Tennessee Certified Beef and other Beef Attributes. J. Agric. Appl. Econ..

[43] Alfnes F., Guttormsen A.G., Steine G., Kolstad K. (2006). Consumers’ Willingness to Pay for the Color of Salmon: A Choice Experiment with Real Economic Incentives. Am. J. Agric. Econ..

[44] Grebitus C., Jensen H.H., Roosen J., Sebranek J.G. (2013). Fresh meat packaging: Consumer acceptance of modified atmosphere packaging including carbon monoxide. J. Food Prot..

[45] Berges M., Casellas K., Rodriguez R., Errea D. (2015). Willingness to pay for quality attributes of fresh beef. Implications on the retail marketing. Microbiology.

[46] García-Torres S., López-Gajardo A., Mesías F.J. (2016). Intensive vs. free-range organic beef. A preference study through consumer liking and conjoint analysis. J. Meat Sci..

[47] Magalhães D.R., Lopes M.A., Rocha C.M. (2016). Socio-economic factors affecting the consumer provision in meat with certification of origin Belo Horizonte, Minas Gerais, Brazil. Arquivos do Instituto Biológico.

[48] Udomkun P., Ilukor J., Mockshell J., Mujawamariya G. (2018). What are the key factors influencing consumers’ preference and willingness to pay for meat products in Eastern DRC?. Food Sci. Nutr..

[49] Yue C.Y., Tong C. (2009). Organic or local? Investigating Consumer Preference for Fresh Produce Using a Choice Experiment with Real Economic Incentives. HortScience.

[50] Van Loo E.J., Caputo V., NaygaJr R.M., Meullenet J.F., Ricke S.C. (2011). Consumers’ willingness to pay for organic chicken breast: Evidence from choice experiment. Food Qual. Prefer..

[51] Azucena G., Loureiro M., Navga R. (2011). Are Valuations from Non hypothetical Choice Experiments Different From Those of Experimental Auctions?. Am. J. Agric. Econ..

[52] Chen X.J., Wu L.H., Xie X.Y. (2018). Assessing the linkages between knowledge and use of veterinary antibiotics by pig farmers in rural China. Int. J. Environ. Res. Public Health.

[53] Blokhuis H.J., Keeling L.J., Gavinelli A., Serratosa J. (2008). Animal welfare’s impact on the food chain. Trends Food Sci. Technol..

[54] Lusk J.L., Tonsor G.T., Schroeder T.C., Hayes D.J. (2018). Effect of government quality grade labels on consumer demand for pork chops in the short and long run. Food Policy.

[55] So Y., Kuhfeld W.F. (1995). Multinomial logit models. Proceedings of the SUGI 20 Conference.

[56] Kuhfeld W.F. (2001). An Introduction to Designing Choice Experiments, and Collecting, Processing and Analyzing Choice Data with SAS.

[57] Rossi P.E., Culloch R.E., Allenby G.M. (1996). The value of purchase history data in target marketing. Mark. Sci..

[58] Adamowicz W., Boxal P., Williams M., Louviere J. (1998). Stated preference approaches for measuring passive use values: Choice experiments and contingent valuation. Am. J. Agric. Econ..

[59] Francesc J., Azucena G. (2017). Does the Valuation of Nutritional Claims Differ among Consumers? Insights from Spain. Nutrients.

[60] De-Magistris T., Lopéz-Galán B. (2016). Consumers’ willingness to pay for nutritional claims fighting the obesity epidemic: The case of reduced-fat and low salt cheese in Spain. Public Health.

[61] Chen Q., Anders S., An H. (2013). Measuring consumer resistance to a new food technology: A choice experiment in meat packaging. Food Qual. Prefer..

[62] Ben-Akiva M., McFadden D., Train K. (2019). Foundations of Stated Preference Elicitation: Consumer Behavior and Choice-based Conjoint Analysis. Found. Trends^®^ Econom..

[63] Train K.E. (2009). Discrete Choice Methods with Simulation.

[64] Ubilava D., Foster K. (2009). Quality certification vs. product traceability: Consumer preferences for informational attributes of pork in Georgia. Food Policy.

[65] Troiano T., Vecchiato D., Marangon F., Tempesta T., Nassivera F. (2019). “Households” Preference for a New ‘Climate-Friendly’ Heating System: Does Contribution to Reducing Greenhouse Gases Matter?. Energies.

[66] Stranieri S., Cavaliere A., Banterle A. (2015). Voluntary Traceability Standards: Which Is the Role of Economic Incentives?.

[67] Lagerkvist C.J. (2013). Consumer preferences for food labelling attributes: Comparing direct ranking and best–worst scaling for measurement of attribute importance, preference intensity and attribute dominance. Food Qual. Prefer..

[68] Curtis K., Dolling M. (2006). The sheep industry-Australia in the global scene. Int. J. Sheep Wool Sci..

[69] Borra D., Tarantola M., Franco A. (2015). Il Consumatore Europeo e il Benessere Animale. Indagine diSlow Food sui Consumi e le Abitudini di Acquisto Della Carne in Funzione Della Percezione Dell’animal Welfare.

[70] Torquati B., Paffarini C., Tempesta T., Vecchiato D. (2019). Evaluating consumer perceptions of social farming through choice modelling. Sustain. Prod. Consum..

[71] Miele M. European Animal Welfare Platform. Report Concerning Consumer Perceptions and Attitudes Towards Farm Animal Welfare. https://www.researchgate.net/publication/267250171_Report_concerning_consumer_perceptions_and_attitudes_towards_farm_animal_welfare.

[72] Swait J. (1994). A Structural Equation Model of Latent Segmentation and Product Choice for Cross-Sectional Revealed Preference Choice Data. J. Retail. Consum. Serv..

[73] Wu L.H., Wang S.X., Zhu D., Hu W.Y., Wang H.S. (2015). Chinese consumers’ preferences and willingness to pay for traceable food quality and safety attributes: The case of pork. China Econ. Rev..

